# Associations between muscle strength and mental health in older adults

**DOI:** 10.1590/1806-9282.20260347

**Published:** 2026-07-20

**Authors:** Ricardo Augusto Leoni de Sousa, Poliany Pereira Cruz, Beatriz Gonçalves de Oliveira, Renato Sobral Monteiro-Júnior, Henrique Nunes Pereira Oliva, Ricardo Cardoso Cassilhas

**Affiliations:** 1 Wright State University, School of Health and Exercise Sciences, Department of Kinesiology and Health – Dayton (OH), United States of America.; 2 Universidade Federal dos Vales Jequitinhonha e Mucuri, Post Graduation Program in Health Sciences (PPGCS) – Diamantina (MG), Brazil.; 3 Wright State University, College of Science and Mathematics, Department of Biological Sciences – Dayton (OH), United States of America.; 4 Universidade Estadual de Montes Claros, Graduate Program of Health Sciences – Montes Claros (MG), Brazil.; 5 Yale University, School of Medicine, Department of Psychiatry – New Haven (CT), United States of America.; 6 Universidade Federal dos Vales Jequitinhonha e Mucuri, School of Biological and Health Sciences (FCBS), Department of Physical Education – Diamantina (MG), Brazil.

## INTRODUCTION

Population aging is a global phenomenon with significant implications for public health systems. According to the World Health Organization (WHO), the number of people aged 60 years or older is expected to double before 2050, reaching approximately two billion worldwide^1^. As life expectancy increases, so does the burden of physical and mental health conditions among older adults^2^. The WHO's World Mental Health Report highlights mental and neurological disorders as major contributors to disability in the elderly, negatively impacting both functionality and overall well-being^1^. In line with this, findings from the Global Burden of Disease Study indicate that depression and anxiety are among the leading causes of years lived with disability in individuals aged 65–84 years^3^.

Mood disorders, such as depression and anxiety, are common among older adults and are characterized by persistent disturbances in emotional states that interfere with daily functioning^4^. Depression is typically marked by sustained sadness, loss of interest or pleasure, fatigue, and cognitive impairments, while anxiety involves excessive worry, restlessness, and physiological arousal^5^. These conditions frequently co-occur, share underlying biological and psychosocial risk factors, and are often underdiagnosed or undertreated in later life due to stigma, atypical symptom presentation, or overlap with physical illness^5,6^. Importantly, mood disorders in older adults are associated with increased healthcare use, diminished quality of life, and higher risk of disability and mortality^2,6^.

Growing evidence points to a bidirectional relationship between mental health and physical function, particularly physical activity and muscle strength^7^. Regular physical activity has been shown to reduce the risk and severity of mood disorders through various mechanisms, including the release of endorphins, reduction of systemic inflammation^8,9^. Muscle strength, as both an outcome of and contributor to physical activity, may reflect not only physiological resilience but also engagement in health-promoting behaviors. Therefore, reduced muscle strength may represent a potential indicator to mood disturbances in older populations.

Muscle strength is a critical component of functional capacity in older adults^10^. It plays a key role in maintaining mobility, independence in daily activities, and in preventing adverse outcomes such as falls, frailty, and mortality^11,12^. The progressive loss of muscle mass and strength, known as sarcopenia, has been consistently linked to an increased risk of functional decline and diminished quality of life^11^.

Given the growing interest in non-pharmacological approaches to mental health in aging populations and the lack of focused evidence syntheses on this topic, particularly from community-based cross-sectional and observational studies, there is a need to investigate these relationships. Thus, this review aimed to evaluate the association between muscle strength and symptoms of depression and/or anxiety in older adults. Importantly, this review is not intended to be systematic, but rather to provide a focused and interpretative synthesis of the current evidence.

## METHODS

This study was designed as a narrative review aimed at providing a focused and integrative overview of the available literature on the association between muscle strength and mental health outcomes in older adults. A literature search was conducted using PubMed, Google Scholar, and LILACS databases without time restrictions. The search strategy combined the following terms: "aging," "muscle strength," "handgrip strength," "depression," "anxiety," and "mental health," using the Boolean operator "AND."

Studies were selected based on their relevance to the topic and their contribution to understanding the relationship between muscle strength and mental health in older populations. Priority was given to observational studies (cross-sectional and cohort), meta-analyses, and key experimental studies that provided mechanistic insights. Two reviewers independently screened titles and abstracts to identify potentially relevant articles, followed by full-text evaluation. Discrepancies were resolved by consensus. As this is a narrative review, no formal risk-of-bias assessment or quantitative synthesis was performed. The objective was to critically interpret and contextualize the existing evidence rather than to provide an exhaustive or systematic synthesis.

## RESULTS AND DISCUSSION

### Association between muscle strength, depressive symptoms and anxiety

A recently published study conducted a meta-analysis focused exclusively on older adults, reporting a weak and negative correlation between handgrip strength and depressive symptoms, although with very high heterogeneity among the included studies^13^. Another recent meta-analysis, which included both adults and older adults, found a lower likelihood of depressive symptoms among individuals with higher muscle strength, while acknowledging substantial variability in populations and measurement methods across studies^14^. Regarding temporality, Huang et al.^15^, through cohort analyses, demonstrated that greater handgrip strength is associated with a reduced subsequent risk of depressive symptoms. As for anxiety, no systematic reviews specifically targeting the association between muscle strength and anxiety symptoms were identified in the consulted literature, highlighting a notable gap in the current body of evidence.

In addition to meta-analytic evidence, several large-scale observational studies have reported consistent associations between lower muscle strength and higher prevalence or incidence of depressive symptoms in older adults^13,15-17^. These findings suggest that the relationship is observable across different populations and settings, although effect sizes are generally modest. Importantly, variability in study design, population characteristics, and measurement tools contributes to heterogeneity and limits direct comparability across studies. It is also important to consider alternative explanations for this association. Depression may lead to reduced physical activity, decreased motivation, and behavioral withdrawal, all of which can contribute to declines in muscle strength over time. Additionally, shared underlying mechanisms, such as chronic low-grade inflammation, endocrine dysregulation, and the presence of comorbid conditions (e.g., cardiovascular disease, diabetes), may independently influence both muscle strength and mental health outcomes. These factors highlight the complexity of the relationship and reinforce the need for cautious interpretation of observational findings.

The inverse association between muscle strength and depressive or anxiety symptoms is biologically and functionally plausible through complementary mechanisms^13,15^. From a functional and social perspective, higher levels of muscle strength are associated with greater autonomy and mobility, and reduced dependence in activities of daily living and instrumental activities of daily living^16^. From a biological standpoint, greater muscle strength in older adults may reflect higher engagement in resistance training and/or lower levels of physical inactivity^18^. Strength training programs have been linked to increased levels of neurotrophic factors, such as brain-derived neurotrophic factors (BDNF) and insulin-like growth factor 1 (IGF-1), as well as enhanced neuroplasticity in hippocampal and prefrontal circuits, and modulation of neurotransmitter systems and the hypothalamic-pituitary-adrenal (HPA) axis^19,20^. These are mechanisms known to play a role in the regulation of mood and anxiety. Anxiety in later life is often underdiagnosed, frequently co-occurs with depression, and may manifest through somatic complaints or agitation rather than typical symptoms^21,22^. Given the shared pathophysiological pathways, such as inflammation, HPA axis dysregulation, and physical inactivity, between anxiety and reduced muscular function, future studies should prioritize investigating this association.

While an inverse relationship between muscle strength and depressive symptoms is consistently noted, the directionality of this association remains uncertain^23^. It is unclear whether reduced muscle strength contributes to depressive symptoms, whether depressive states reduce motivation for physical activity and muscle maintenance, or whether both phenomena share common underlying biological mechanisms. Longitudinal studies are needed to clarify temporal relationships and potential bidirectional influences.

Moreover, the heterogeneity in assessment tools for both muscle strength and mental health outcomes adds complexity to the interpretation of findings^24^. While handgrip strength is widely used as a simple and reliable proxy for overall muscle strength^23,24^, it may not fully capture the multifaceted nature of muscular fitness. Likewise, depressive and anxiety symptoms are usually assessed using different scales across studies, potentially introducing variability in symptom severity categorization and limiting direct comparisons.

Despite these methodological differences, the repeated observation of a negative association between muscle strength and depressive symptoms across diverse populations lends some robustness to the findings^14^. Even when the strength of correlation is modest, the consistency of direction suggests an underlying relationship that merits further exploration^14,23,24^. Studies to be conducted in low- and middle-income countries may help elucidate how socioeconomic and cultural factors mediate this association.

Additionally, depression in older adults often has a distinct clinical presentation compared to younger populations and across different species, sometimes overlapping with apathy, fatigue, or cognitive decline and experimental studies, including animal models, provide mechanistic support^25,26^. This raises the question of whether low muscle strength may serve not only as a marker of physical frailty but also as a red flag for subclinical or atypical depressive states. Screening older adults using muscle strength tests may have potential utility as a complementary indicator fit by identifying those at risk of physical decline and those who might benefit from mental health evaluation.

**Figure 1:**
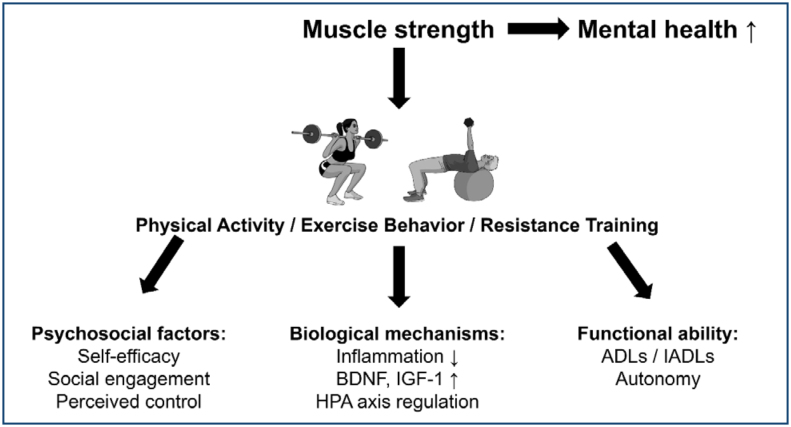
Conceptual model illustrates hypothesized pathways linking muscle strength and mental health in older adults. The proposed mechanisms are based on existing literature but have not been directly tested within a single empirical framework and should be interpreted as theoretical. Muscle strength may influence mental health directly and indirectly through a range of biological, functional, and psychosocial mechanisms. Key mediating pathways include increased physical activity through changes in exercise behavior and incorporation of resistance training as part of the physical training, which in turn lead to improved functional capacity (e.g., autonomy in daily activities), greater self-efficacy and social engagement, reduced systemic inflammation, enhanced neuroplasticity via BDNF and IGF-1 increasement, and management of HPA axis. Finally, all these changes through improvement in muscle strength will contribute to better mental health.

The conceptual framework (Figure 1) illustrates potential pathways linking muscle strength to mental health outcomes in older adults. These pathways are based on existing biological and functional evidence. However, they remain hypothetical and should be interpreted as a model to guide future research rather than as a confirmed causal structure.

## CONCLUSION

This narrative review highlights a consistent inverse association between muscle strength, particularly handgrip strength, and depressive symptoms in older adults. However, the magnitude of this association is generally modest, and substantial heterogeneity exists across studies. Importantly, the direction of causality remains unclear, and current evidence is insufficient to support the use of muscle strength as a standalone screening tool for mental health outcomes.

While muscle strength assessment is simple, objective, and clinically accessible, its role in mental health evaluation should be considered exploratory and complementary. Future research, particularly longitudinal studies and randomized controlled trials, is needed to clarify causal mechanisms and determine whether muscle-strengthening interventions can effectively improve mental health outcomes in aging populations.

## Data Availability

The datasets generated and/or analyzed during the current study are available from the corresponding author upon reasonable request.
